# A Cadaveric Case of a Circular Torcular Herophili

**DOI:** 10.7759/cureus.3099

**Published:** 2018-08-04

**Authors:** Sasha Lake, Juan J Altafulla, Joe Iwanaga, Rod J Oskouian, Marios Loukas, R. Shane Tubbs

**Affiliations:** 1 Anatomical Studies, St. George's, St. George, GRD; 2 SNI, Seattle Science Foundation, Seattle, USA; 3 Medical Education and Simulation, Seattle Science Foundation, Seattle, USA; 4 Neurosurgery, Swedish Neuroscience Institute, Seattle, USA; 5 Anatomical Sciences, St. George's University, St. George's, GRD; 6 Neurosurgery, Seattle Science Foundation, Seattle, USA

**Keywords:** waiting, torcular herophili, confluence sinus, torcular morphology, torcular variants

## Abstract

The torcular Herophili is formed by the joining of the straight sinus, superior sagittal sinus, and transverse sinus. The anatomic configuration of the torcular Herophili is highly variable. In the current literature, classification systems define up to nine subtypes of the torcular Herophili. The frequency of prevalence of these anatomical variants is also variable. Herein is a case report of a circularly-shaped torcular Herophili found during cadaveric dissection.

## Introduction

The confluence of sinuses also called the torcular Herophili lies near the internal occipital protuberance and receives venous drainage from various regional dural venous sinuses [1]. Classically, the torcular forms at the convergence of the superior sagittal, straight, and transverse sinuses. However, the anatomical morphology of the torcular is highly variable, and the classification systems thus far describe nine subtypes of torcular morphology. The classical form of the torcular represents the most common subtype [2-3]. Other subtypes are under-reported throughout the literature and can have clinical/surgical implications [4].

## Case presentation

**Figure 1:**
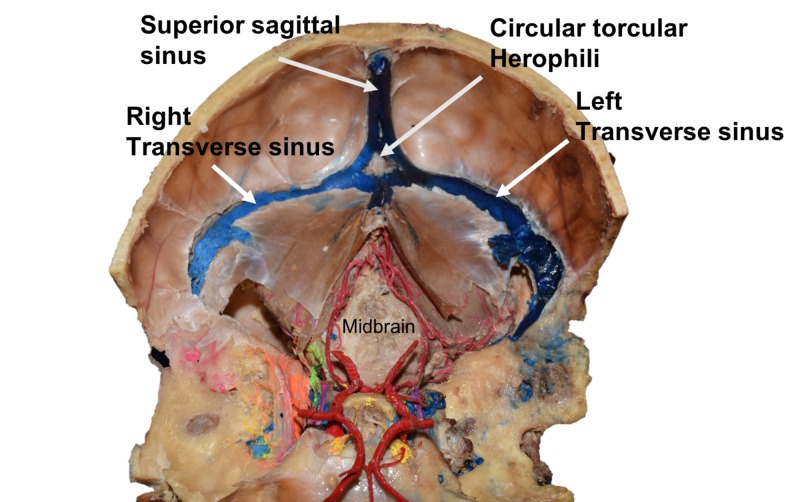
Internal view of the case presented herein Note the circular torcular Herophili and its relationship to the transverse and superior sagittal sinuses.

During the routine dissection of an adult male cadaver aged 87 years at death, an unusual arrangement of the dural venous sinuses was identified (Figure 1). This specimen had previously undergone blue latex injection of the intracranial venous system. Posteriorly, the region overlying the internal occipital protuberance was found to be encircled by a splitting of the superior sagittal sinus above into two more or less equal parts each draining into one of the transverse sinuses. Additionally, there was a midline connection between the left and right transverse sinuses, effectively forming a venous circle around the internal occipital protuberance, i.e., a circular torcular Herophili. No other neurovascular variations were identified in this specimen.

## Discussion

Recently, Matsuda et al. reexamined anatomical variations of the torcular, focusing on venous flow and the continuity of the superior sagittal and transverse sinuses. They reported that venous flow from the superior sagittal sinus to the transverse sinus could be either symmetric or asymmetric [5]. The findings of the Matsuda et al. study added to the literature compilation that established venous drainage at the torcular as symmetric or asymmetric [1-2,4,6-7]. Despite the asymmetry of venous flow, on venography, the torcular usually forms an inverted T shape—with the superior sagittal sinus representing free communication between the superior sagittal, the straight, the occipital, and the transverse sinuses [6]. The inverted T shape accurately describes the traditional anatomy and the most common depiction of the torcular [3]. However, the literature also reported many anatomical variations of the torcular [1-3,5-6,8]. Kobayashi et al. elaborately classified nine types of variants of the torcular. Their study further classed communications between the right and left transverse sinus into four subtypes [3].

Recently, a circular variant of the torcular was reported on magnetic resonance imaging from a patient suffering from chronic headaches and questionable papilledema [4]. In the present case, a similar finding was seen in a cadaveric dissection. Another interpretation of the present case could also be a splitting of the superior sagittal sinus into left and right parts that travel to the left and right transverse sinuses, respectively. This, coupled with a communication across the midline of the left and right transverse sinuses, resulted in a circular configuration in the region of the torcular. The development of the torcular occurs during the fourth to sixth months of gestation [8-10]. A series of transitional growth and regression patterns of primitive dural plexuses to dural sinuses occurs during this period. Irregular patterns of growth can lead to different heights and size asymmetry of the dural sinuses, mild to marked irregularities, or even an absence of the medial portion of the transverse sinuses [8].

## Conclusions

The embryology of the dural sinuses further elucidates the genesis of anatomical variants. The torcular Herophili is an area of interest for neurosurgical and interventional procedures. Given the high variability of the region, an awareness of normal anatomy and variations such as seen in the case presented herein is crucial for preoperative planning and during the interpretation of cranial imaging.
